# Supplementary material for: Meta-Inflammation and De Novo Lipogenesis Markers Are Involved in Metabolic Associated Fatty Liver Disease Progression in BTBR ob/ob Mice
Source: Int J Mol Sci. 2022 Apr 2;23(7):3965. doi: 10.3390/ijms23073965 (PMC8999923; doi:10.3390/ijms23073965)
Supplement: Supplementary file 1 [file ijms-23-03965-s001.zip › ijms-1623202-supplementary.pdf]

International Journal of
Molecular Sciences

MDPI

Article

Meta-Inflammation and De Novo Lipogenesis Markers Are Involved in Metabolic Associated Fatty Liver Disease Progression in BTBR ob/ob Mice

Supplementary Table S1. Primers for PCR detection.

Target gene	TaqMan Assay	Target gene	TaqMan Assay
Tnf (TNF-α)	Mm00443258_m1	Ifng (IFN-γ)	Mm01168134_m1
IL12	Mm00434169_m1	IL15	Mm00434210_m1
Ccl2	Mm00441242_m1	Ccl5	Mm01302428_m1
Cxcl10	Mm00445235_m1	Cx3cl1	Mm00436454_m1
Tlr4	Mm00445273_m1	Tgfb1 (TGF-β1)	Mm01178820_m1
Ctgf	Mm01192933_g1	Hmgcr	Mm01282499_m1
Soat1	Mm00486279_m1	Acaca (Acc1)	Mm01304289_m1
Fasn	Mm00662319_m1	Scd1	Mm00772290_m1
Dgat2	Mm00499536_m1	Ppara (PPAR-α)	Mm00440939_m1
Pparg (PPAR-γ)	Mm00440940_m1	Mlxipl (Chrebp1)	Mm00498811_m1
Srebf1 (Srebp1)	Mm00550338_m1	Ppargc1a (PGC-1α)	Mm01208835_m1
Dnm1l (Drp1)	Mm01342903_m1	Msr1(Cd204)	Mm00446214_m1
Nfe2l2 (Nrf2)	Mm00477784_m1	Hmox1	Mm00516005_m1
Sod1	Mm01344233_g1	Catalase	Mm00437992_m1
Slc27a2 (Fatp2)	Mm00449517_m1	Abca1	Mm00442646_m1
Abcg1	Mm00437390_m1	18S (VIC)	4310893E

Gene SYBR Green	Forward sequence (5′-3′)	Reverse sequence (5′-3′)
CD36	GAACCACTGCTTTCAAAAACTGG	TGCTGTTCTTTGCCACGTCA
Grem1	GTGACAGAATGAATCGCACCG	CTTGTCAGGAGGCGGAATGG

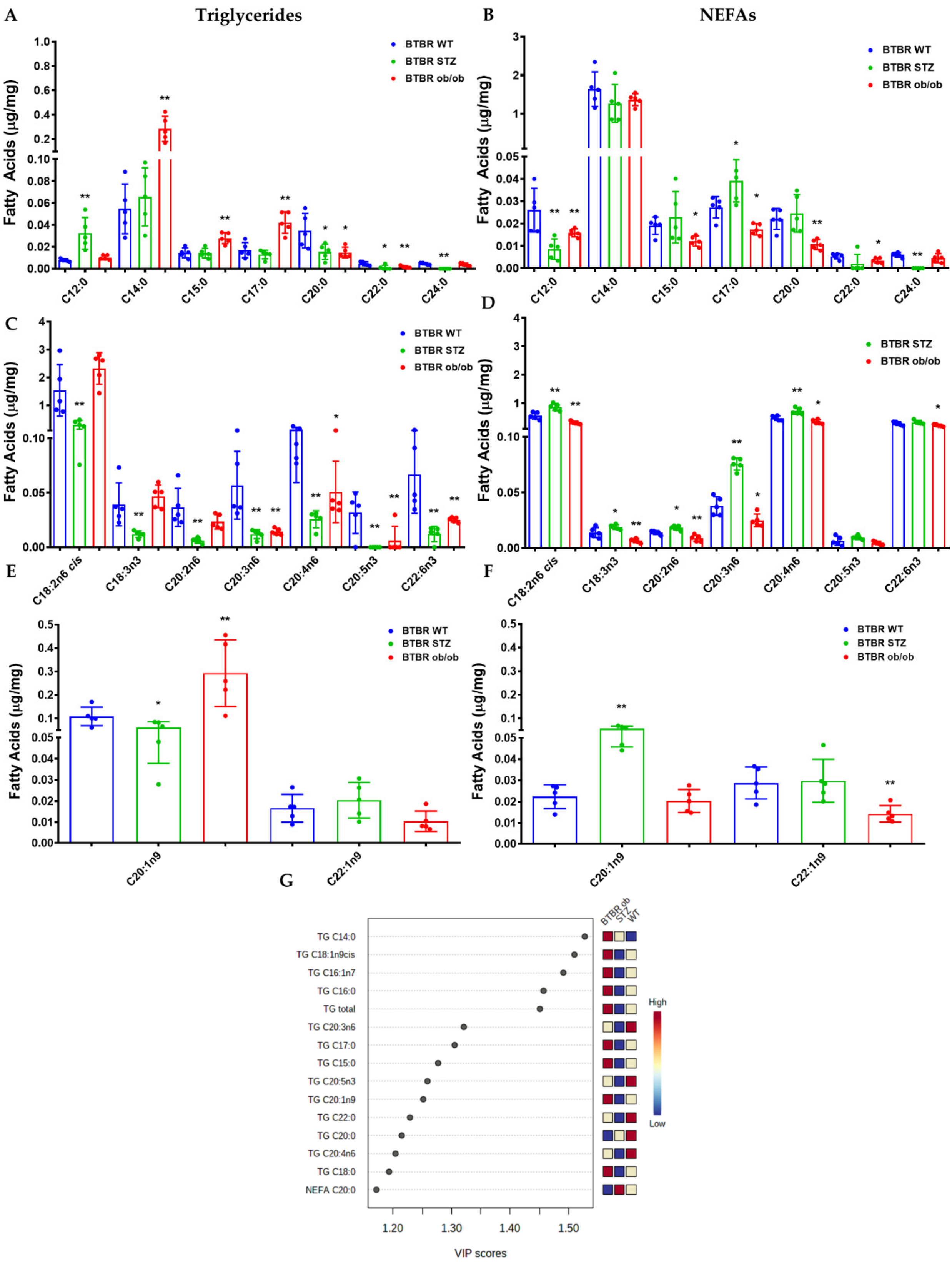

Supplementary Figure S1. Detailed quantification of major fatty acid components from triglycerides (left) and NEFAs (right) in liver. (A) and **(B)** saturated fatty acids; **(C)** and **(D)** polyunsaturated fatty acids (PUFA); and **(E)** and **(F)** monounsaturated fatty acids (MUFA) **(G)** Variable Importance in Projection (VIP) involved in oPLS discriminant

analysis. Data are shown as scatter dot plots and mean ± SEM of each group (n=6 mice/group); *$p < 0.05$, ** p <0.01, vs. BTBR WT.

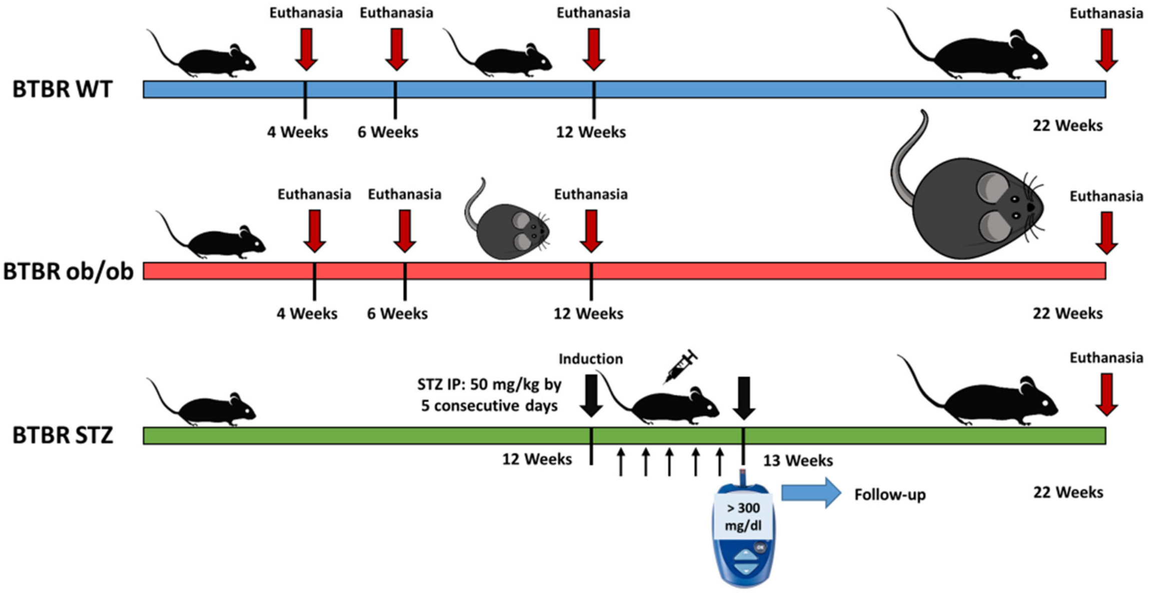

Supplementary Figure S2. Experimental design. BTBR wild type (WT) and leptin-deficient (ob/ob) mice at 4, 6, 12 and 22 weeks-old were sacrificed (euthanized). BTBR WT at 12 week-old mice were injected with STZ low-doses (50 mg/kg) for 5 consecutive days by intraperitoneal pathway. During the first week post-STZ injection, mice with glycemias ≥ 300 mg/dl every week until 22 weeks-old were sacrificed and included in the study.
